# Itch in recessive dystrophic epidermolysis bullosa: findings of PEBLES, a prospective register study

**DOI:** 10.1186/s13023-023-02817-z

**Published:** 2023-08-09

**Authors:** Jemima E. Mellerio, Elizabeth I. Pillay, Lesedi Ledwaba-Chapman, Alessandra Bisquera, Susan J. Robertson, Marieta Papanikolaou, John A. McGrath, Yanzhong Wang, Anna E. Martinez, Eunice Jeffs

**Affiliations:** 1 St John’s Institute of Dermatology, Guy’s and St Thomas’ NHS Foundation Trust, Westminster Bridge Road, London, SE1 7EH UK; 2 Genetic Skin Disease Group, King’s College London, London, UK; 3 Department of Population Health Sciences, King’s College London, London, UK; 4 Departments of Dermatology, The Royal Children’s Hospital, The Royal Melbourne Hospital and Monash Health, Melbourne, Australia; 5 Department of Dermatology, Great Ormond Street Hospital for Children NHS Foundation Trust, London, UK

**Keywords:** Epidermolysis bullosa, Itch, Natural history, Quality of life, Disease severity

## Abstract

**Background:**

Itch is common and distressing in epidermolysis bullosa (EB) but has not previously been studied in depth in different recessive dystrophic EB (RDEB) subtypes.

**Objectives:**

As part of a prospective register study of the natural history of RDEB we explored features of itch, medications used, and correlation with disease severity and quality of life.

**Methods:**

Fifty individuals with RDEB aged 8 years and above completed the Leuven Itch Scale (LIS) (total 243 reviews over a 7-year period). Data included itch frequency, severity, duration, distress, circumstances, consequences, itch surface area and medications for itch. The iscorEB disease severity score and the validated EB quality of life tool, QOLEB, were compared to LIS domains and analysed by RDEB subtype.

**Results:**

Itch was frequent, present in the preceding month in 93% of reviews. Itch severity and distress were significantly greater in severe (RDEB-S) and pruriginosa (RDEB-Pru) subtypes compared to intermediate RDEB (RDEB-I). Itch medications were reported in just over half of reviews including emollients, topical corticosteroids and antihistamines; the proportion of participants not using medication despite frequent pruritus suggests limited efficacy. In inversa RDEB (RDEB-Inv) and RDEB-I, LIS domains correlated with iscorEB and QOLEB. In contrast to previous studies, correlations were lacking in RDEB-S suggesting that global disease burden relatively reduces the contribution of itch.

**Conclusions:**

This comprehensive study of RDEB-associated itch highlights differences between RDEB subtypes, suggests an unmet need for effective treatments and could serve as control data for future clinical trials incorporating itch as an endpoint.

**Supplementary Information:**

The online version contains supplementary material available at 10.1186/s13023-023-02817-z.

## Background

Epidermolysis bullosa (EB) is a heterogeneous group of inherited mucocutaneous fragility disorders. Four major types of EB are differentiated by their ultrastructural plane of skin separation: EB simplex (EBS), junctional EB (JEB), dystrophic EB (DEB) and Kindler EB (KEB) [1].

Recessive DEB (RDEB) encompasses a number of distinct subtypes that all result from biallelic mutations in *COL7A1*, the gene encoding type VII collagen, the major component of anchoring fibrils at the dermal-epidermal junction [2, 3]. Severe RDEB (RDEB-S) is associated with marked mucocutaneous blistering, chronic wounds, scarring, impaired nutrition and growth, anaemia and, among other complications, development of aggressive cutaneous squamous cell carcinomas (SCCs), the main cause of death in early adulthood [4–7]. Intermediate RDEB (RDEB-I) presents with less severe generalized skin fragility and scarring, also with internal complications and SCC but occuring later and less frequently [4, 5, 7]. Inversa RDEB (RDEB-Inv) usually manifests as generalized blistering early in life but, over time, develops a predilection for flexural sites such as axillae, groins and genitals, with marked intra-oral and oesophageal involvement [8]. The rare pruriginosa form is usually dominantly inherited but can be recessive (RDEB-Pru) [9]. Individuals progressively develop extremely itchy skin with characteristic prurigo-like nodules or plaques, usually starting on the lower legs but with more generalized spread in some cases.

Itch is common in all forms of EB [10–12] and causes significant distress [11,13−16]. Pruritus in RDEB tends to be more severe than in other EB types [11] with similar intensity to itch associated with atopic dermatitis, chronic urticaria and nodular prurigo [13]. Factors that exacerbate EB itch include healing of wounds, skin infections, dry skin, heat, sweating and stress [11, 14, 15]. EB-associated itch negatively impacts on quality of life and sleep, [11, 14] and scratching contributes to skin damage [16]. Interventions include emollients, topical corticosteroids, antihistamines, neuropathic pain medicines and neurokinin-1 receptor antagonists although these generally have limited efficacy [12, 17, 18].

The precise mechanisms of EB pruritus are poorly understood but are likely multifactorial including acute and chronic wounds, dysregulated inflammation, impaired skin barrier function and a possible neural mechanism [19–24]. Varied aetiology may partly explain the poor responsiveness of EB itch to standard treatments [11]. This, in conjunction with distress caused by itch and the consequences of scratching worsening blistering, highlights the need for further studies into pathomechanisms of EB-associated itch and development of effective therapies.

The Prospective Epidermolysis Bullosa Longitudinal Evaluation Study (PEBLES) is a register study which has recruited children and adults with RDEB from the United Kingdom London EB reference centres since late 2014. It records detailed participant information including subjective, objective, laboratory and health economic data. The aim of PEBLES is to delineate, in detail, the natural history of different RDEB subtypes to inform prognostication and identify robust and meaningful endpoints for future clinical trials. Here, we present PEBLES data relating to itch and the impact it has on individuals with RDEB.

## Materials and methods

### Study population

This cohort study included PEBLES participants attending either Great Ormond Street Hospital for Children (children) or Guy’s and St Thomas’ Hospital (adults). RDEB diagnosis was confirmed by skin biopsy and/or genetic testing and classified to subtype according to clinical features. Participants were recruited between 19th November 2014 and 17th November 2021 with reviews conducted annually for those 10 years and older or 6-monthly in under-10s. At each visit, information was collected on EB- and non-EB-related health issues, procedures and complications, disease severity scores, subjective data including itch, pain and quality of life, laboratory and imaging reports and information relating to costs of care. At subsequent reviews, information was updated since the preceding visit. Data were pseudonymised (date of birth retained to link participants’ age to reviews) and recorded in a Research Electronic Data Capture (REDCap) database. PEBLES was ethically approved by the UK Research Ethics Committee and Health Research Authority (IRAS 142,032).

### Measures

The Leuven Itch Scale (LIS) (version 1.0 and 2.0 US English) is a self-reported measure validated for adults aged 18 and over which captures different aspects of itch over the preceding month including location, sensory perceptions and management of itch [25]. Algorithms convert raw scores into 6 sub-score scales from 0 to 100 for itch frequency, severity, duration, distress, consequences and surface area where higher scores reflect greater itching. The LIS has been validated in different skin disorders including atopic dermatitis, chronic urticaria and burns, [25] and has been used in a prior study of itch in different types of EB [14]. We offered children aged 8 and above the opportunity to complete LIS, as there is no suitable comprehensive alternative, and report their findings both with and separate to the adult scores. Children, mostly with RDEB-S, and their parents were eager to report their experience of itch and reported no difficulty in completing the questionnaires.

At each review participants completed the LIS and reported medication; these were compared to identify relevant treatments that were not reported as itch management. EB severity was recorded using the validated iscorEB tool, which comprises clinician and patient scores with one item asking participants to rate itch over the previous 4 weeks from none (0) to worst possible (8) [26]. Adults also completed the QOLEB quality of life questionnaire which is validated for individuals with EB aged 18 years and above [27].

### Statistical analysis

Results were reported for all participant reviews with complete data and separately for index review, that is, the first review for each participant with complete information on for all 6 LIS subscales. Findings are reported for all RDEB and by subtype at index review, with some findings separately reported for under 18 years, and results for all reviews provided as supplementary tables.

Index review characteristics were summarised using counts and percentages. As per the LIS manual, results were expressed as the mean (standard deviation; sd). Pairwise comparisons between RDEB subtypes for different LIS parameters were computed using the Mann-Whitney U test. Statistical significance is defined at p < 0.05 and was not adjusted for multiplicity, however the number of significant results that are expected to occur by chance are stated. Correlations between total iscorEB scores and QOLEB scores with LIS sub-scores were calculated using Spearman’s rank correlation. All analyses were performed using R v4.1.3.

## Results

### Characteristics of itch in RDEB

Fifty individuals aged over 8 years completed the LIS: 20 with RDEB-S, 18 with RDEB-I, 9 with RDEB-Inv and 3 with RDEB-Pru (Table 1). These participants contributed a total of 243 reviews, median 5 reviews (range 1–7) of which 31 reviews from 8 participants (7 with RDEB-S and 1 with RDEB-I) were provided when under 18 years.

**Table 1:** Demographics of the study group. Results are presented as n (%)

			RDEB subtype			
Characteristic	Category	Overall	RDEB-S	RDEB-I	RDEB-Inv	RDEB-Pru
n		50	20	18	9	3
Age group	8–17	8 (16)	7 (35)	1 (6)	0 (0)	0 (0)
	18–39	21 (42)	10 (50)	3 (17)	6 (67)	2 (67)
	40+	21 (42)	3 (15)	14 (78)	3 (33)	1 (33)
Gender	Female	29 (58)	12 (60)	11 (61)	6 (67)	0 (0)
	Male	21 (42)	8 (40)	7 (39)	3 (33)	3 (100)
All ethnicities	White British	37 (74)	12 (60)	15 (83)	7 (78)	3 (100)
	Irish	1 (2)	0 (0)	1 (6)	0 (0)	0 (0)
	Other White	5 (10)	4 (20)	0 (0)	1 (11)	0 (0)
	White and Asian	1 (2)	1 (5)	0 (0)	0 (0)	0 (0)
	Indian	2 (4)	2 (10)	0 (0)	0 (0)	0 (0)
	Pakistani	3 (6)	1 (5)	1 (6)	1 (11)	0 (0)
	Any other ethnic group	1 (2)	0 (0)	1 (6)	0 (0)	0 (0)

**Fig. 1:**
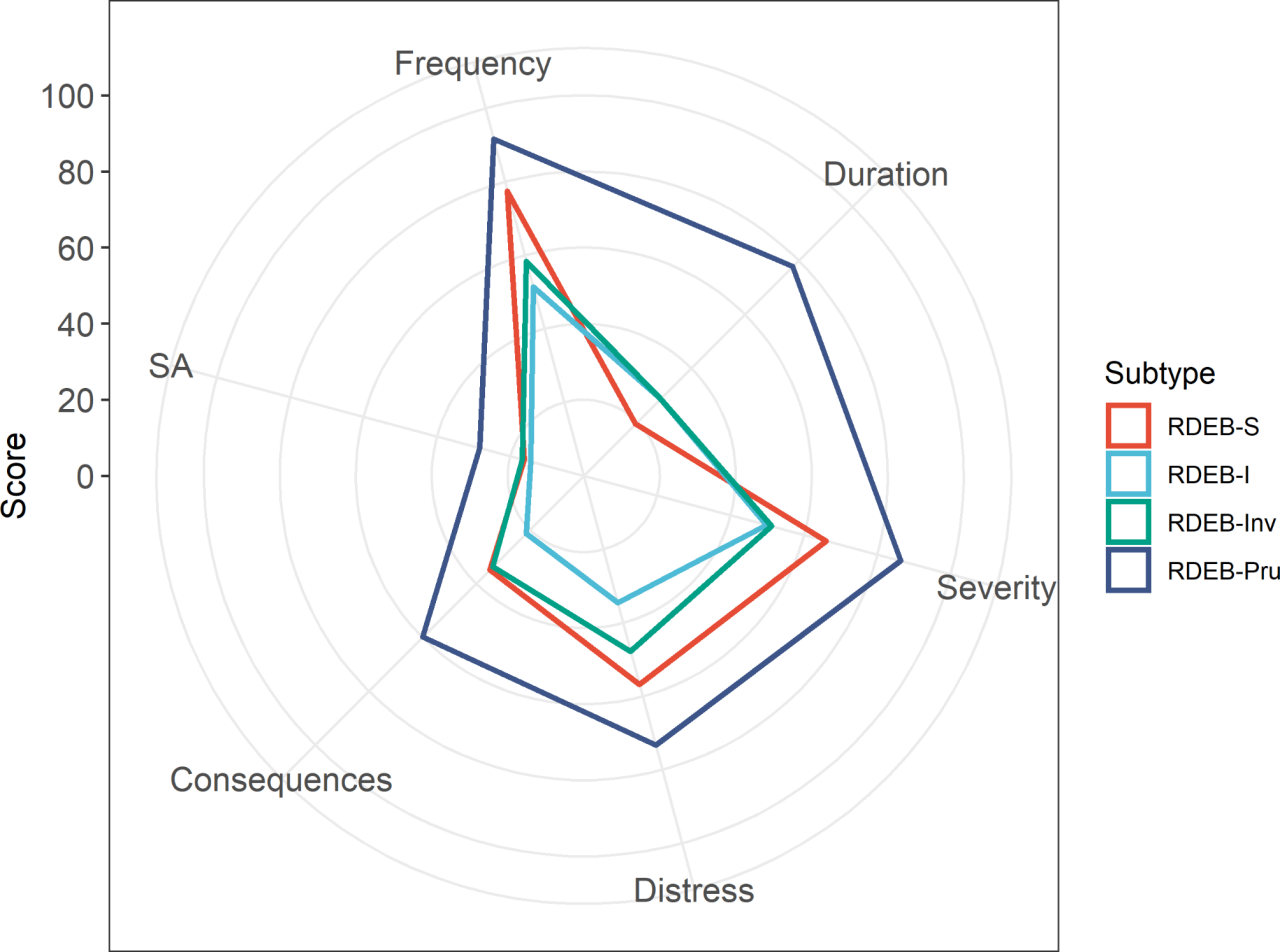
Radar graph displaying mean LIS metrics at index review by RDEB subtype

**Fig. 2:**
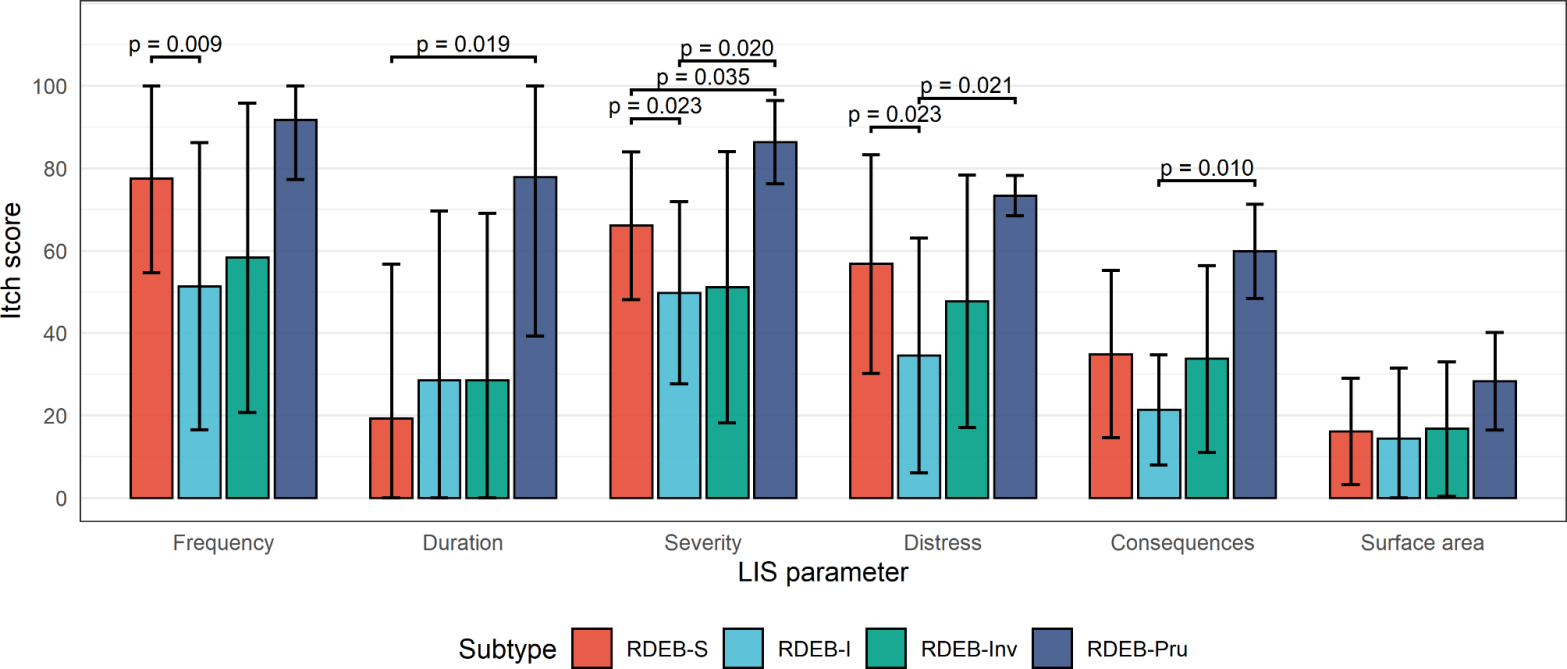
Bar plot displaying mean LIS metrics by RDEB subtype with pairwise comparisons. Error bars indicate the standard deviation. Only significant (P < 0.05) differences are displayed. As we have not adjusted for multiple comparisons, we would expect two significant results to occur by chance

Index review LIS itch sub-scores by RDEB subtype are shown in Table 2; Fig. 1. Pairwise comparisons between RDEB subtypes are shown in Fig. 2 and Additional file 1.

**Table 2:** Mean LIS itch score by RDEB subtype at index review (n = 50). Index review is the first review for each participant with complete information on for all 6 LIS subscales. Results are presented as mean (sd)

		RDEB subtype			
Itch score	Overall	S	I	Inv	Pru
n	50	20	18	9	3
Frequency	66 (32)	78 (23)	51 (35)	58 (38)	92 (14)
Duration	28 (40)	19 (37)	29 (41)	29 (40)	78 (38)
Severity	60 (24)	66 (18)	50 (22)	51 (33)	86 (10)
Distress	49 (29)	57 (27)	35 (28)	48 (31)	73 (5)
Consequences	32 (20)	35 (20)	21 (13)	34 (23)	60 (11)
Surface area	17 (15)	16 (13)	14 (17)	17 (16)	28 (12)

#### Itch frequency

Participants with RDEB-Pru and RDEB-S reported the greatest mean (sd) frequency at index LIS (Table 2). Itch frequency in RDEB-S was significantly greater than in RDEB-I (p = 0.009). Seven participants reported no itch in the month prior to index review (1 RDEB-S, 4 RDEB-I, 2 RDEB-Inv).

**Fig. 3:**
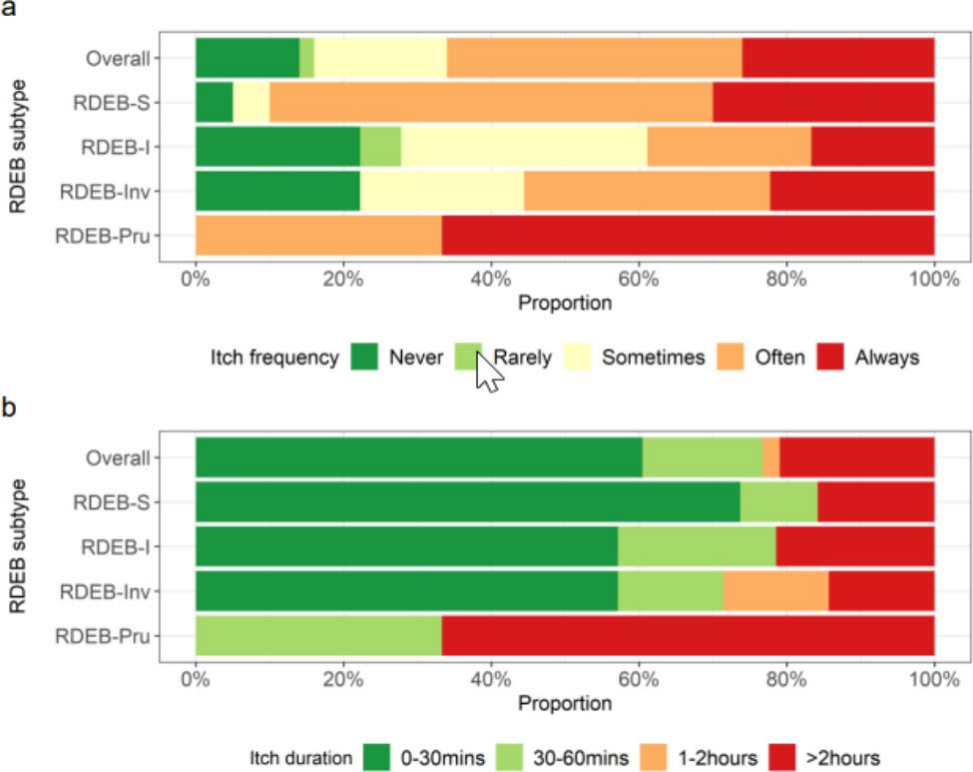
**(a)** Stacked bar plot displaying the distribution of itch frequency within each RDEB subtype (n = 243 from 50 participants). Never: no itch experienced in the preceding month; Rarely: itch 1 to a few times per month; Sometimes: itch 1 to a few times per week; Often: itch 1 to a few times per day; Always: constant itch. **(b)** Stacked bar plot displaying the distribution of itch duration within each RDEB subtype (n = 226 from 48 participants). Itch duration refers to the average length of itching episodes in the preceding month

Of the 243 reviews from all time-points, 16 (7%) reported no itch in the preceding month (1 RDEB-S, 9 RDEB-I (4 participants), 6 RDEB-Inv (2 participants)) and were therefore excluded from analysis for those reviews; only 2 patients (9 reviews) reported no itch at each of their reviews. Itch was experienced ‘always’ or ‘often’ at 89/102 (87%) RDEB-S reviews, 36/85 (42%) RDEB-I reviews, 26/46 (57%) RDEB-Inv reviews and 10/10 (100%) RDEB-Pru reviews (Fig. 3a). Itch frequency appeared to decrease with age in RDEB-I and RDEB-Inv but remained more constant in RDEB-S (Additional file 2a).

#### Itch duration

Participants with RDEB-S, RDEB-I and RDEB-Inv had similar itch duration at index review with most having durations of up to 30 min although transformed scores showed that RDEB-S had the shortest duration of itch (Table 2). In contrast, RDEB-Pru was associated with longer itch duration; two reviews reported a duration of over 2 h per episode of itch. Itch duration was significantly less for RDEB-S compared to RDEB-Pru (p = 0.019) (Fig. 2). Data from all reviews (n = 226, 48 participants) also reflected greater itch duration in RDEB-Pru (Fig. 3b) and a tendency for younger RDEB-S participants to have shorter itch duration than older RDEB-S and RDEB-I participants (Additional file 2b).

#### Itch time period

Itch was reported at index review as most frequent at night-time (77%) and least frequent in the morning (37%), with the exception of RDEB-I patients who experienced most itch in the day (64%) and least in the morning (47%) (Additional file 3). The time period when itch was experienced was available for 227 reviews (48 participants) with a similar pattern to index reviews.

#### Itch severity

Mean (sd) itch severity scores at index review are shown in Table 2. We found greater itch severity in RDEB-S compared to RDEB-I (p = 0.023), although no difference in adult and child scores, and greater itch in RDEB-Pru compared to RDEB-S (p = 0.035) and RDEB-I (p = 0.020) (Fig. 2). Similar results were seen in the 225 reviews (43 participants) with itch severity data (Additional file 2c).

#### Itch distress

Mean (sd) distress at index review is shown in Table 2. On average, participants with RDEB-S and RDEB-Pru reported significantly higher itch distress scores than those with RDEB-I (p = 0.023 and p = 0.021, respectively) (Fig. 2). Results from all reviews with itch distress data (n = 224, 48 participants) suggested greater distress in younger participants but this may reflect the greater representation of RDEB-S in this group (Additional file 4a).

#### Itch consequences

Index review mean (sd) consequence scores are shown in Table 2. There was a significantly higher mean itch consequences score in RDEB-Pru compared to RDEB-I (p = 0.010) (Fig. 2). Data on consequences for all reviews (n = 223, 48 participants) are shown by RDEB subtype and age group in Additional file 4b and Additional file 5.

#### Itch circumstances

The circumstances associated with greatest itch occurrence for all RDEB at index review were being in a hot environment (60%), feeling stressed out (53%) and sweating (47%) (Additional file 3). Individuals with RDEB-S scored particularly highly in these circumstances with itch occurrence associated in 79%, 74% and 63%, respectively. In contrast, itch occurrence was low overall when in a cold environment (14%) and on contact with air (19%). Findings on itch circumstances for all reviews (n = 227, 48 participants) were similar to index review except more reported itch on contact with air (31%); (Additional file 6).

#### Itch characteristics

Overall, itching at index review manifested mostly as a tickling sensation (69%) but only rarely as tingling (19%) or stinging (14%) (Additional file 3). This pattern was seen in all RDEB subtypes except for those with RDEB-Pru who reported they were more likely to experience a burning sensation (67%). Findings for all reviews (n = 227, 48 participants) were similar except more reported a tingling sensation (30%) (Additional file 7).

#### Itch surface area

There were no differences between subgroups in mean itch surface area (ISA) at index review (Table 2; Fig. 2). Additional file 4b shows the majority of scores within each subtype (165 reviews, 47 participants) were between 0 and 20 with few > 40, suggesting data are likely skewed to the left. However, 62 (27%) reviews were missing information on at least one element of the ISA score so could not be included in this analysis.

#### Treatments for itch

Of 50 index reviews, 12 (24%) participants used emollients (9 as monotherapy); two (4%) used menthol-containing emollient and one (2%) used a topical corticosteroid for itch. Fourteen participants (28%) used oral antihistamines, 11 as monotherapy and 7 took two or more antihistamines. The treatments used by RDEB subtypes are shown in Table 3 and Additional file 8. Of 243 reviews with a valid LIS 148 (61%) reported using medication for itch demonstrating variable usage over time. Of 36 participants who reported not using an ointment to treat their itch in their index LIS, 14 (39%) reported using an emollient or topical corticosteroid on PEBLES medication review. Thirty-six participants reported not using medication for itch, none of whom reported antihistamines on their medication review. Satisfaction with itch medication from the LIS showed a mean (sd) at index review for all RDEB of 41.3 (31.3) (out of 100), with similar scores for all RDEB subtypes and when all reviews were considered (Additional file 9).

**Table 3:** Treatment use by subtype (n = 50). Results are presented as x/n (%), where x is the number of participants reporting use and n is the total number of participants. Only the index review LIS of each participant is considered

Treatment	Overall	RDEB-S	RDEB-I	RDEB-Inv	RDEB-Pru
Emollient without menthol	12/50 (24)	4/20 (20)	3/18 (17)	3/9 (33)	2/3 (67)
Emollient with menthol	2/50 (4)	0/20 (0)	1/18 (6)	1/9 (11)	0/3 (0)
Corticosteroid	1/50 (2)	1/20 (5)	0/18 (0)	0/9 (0)	0/3 (0)
Antihistamine	14/50 (28)	9/20 (45)	2/18 (11)	1/9 (11)	2/3 (67)

#### iscorEB and itch

Correlations between iscorEB itch scores and LIS itch frequency and severity were calculated by RDEB subtype (Additional file 10). There was a moderate (r = 0.4–0.6) relationship between itch frequency and iscorEB itch score for RDEB-I patients.

For all reviews total iscorEB score correlated strongly (r = 0.6–0.8) with LIS itch frequency, distress, severity and consequences in RDEB-I, and correlated strongly or very strongly (r > 0.8) with all LIS itch domains in RDEB-Inv (Additional files 11 and 12). Correlations in RDEB-S were weak or negligible for all LIS domains with the exception of ISA which had a moderate correlation with total iscorEB score.

#### QOLEB and itch

Correlations between total QOLEB scores and different domains of the LIS for all adult reviews are shown by RDEB subtype (Additional files 13 (index reviews only) and 14 (all reviews)). For all reviews, correlations for RDEB-Inv were strong between total QOLEB and LIS itch severity, distress, consequences and ISA, and moderate for RDEB-I between total QOLEB and LIS itch frequency, distress, consequences and ISA. In contrast, correlations in RDEB-S were weak or negligible for all LIS domains except ISA.

## Discussion

In our study, we used the LIS to explore different elements of itch in a cohort of 50 participants with RDEB aged 8 years and above. To our knowledge, this is the second and largest study using the LIS in EB [14].

Itch frequency in our cohort was high with occurrence in the preceding month in 227 (93%) of 243 reviews from 50 participants. This concurs with previous studies that demonstrated frequent self-reported itch in 85% (n = 83), [10] 100% (n = 6) [14, 28] and 100% (n = 5) [11] of individuals with RDEB, although these studies did not break down by RDEB subtype [11, 14, 15, 28].

Our finding that participants with RDEB-Pru reported itch episode duration of over 2 h in almost half of reviews supports the clinical phenotype of RDEB-Pru where greater itch duration causes increased scratching and resultant lichenified prurigo-like skin changes. The shorter itch duration in RDEB-S participants may reflect that they tend to have more skin wounds and therefore more pain, which may be a more prevalent or distressing symptom, thereby reducing the relative perception of itch.

Itch severity and distress were high across all RDEB groups and significantly greater in RDEB-S compared to RDEB-I (p = 0.023 and p = 0.023, respectively). Although previous studies have highlighted correlations between EB severity and pruritus and that EB-associated itch is distressing for patients, [11,13−15] our study is the first to quantify this in different RDEB subtypes. Consequences of itch were greatest in RDEB-Pru likely reflecting the itch severity, frequency and duration in this group.

Circumstances associated with high itch occurrence included being in a hot environment and when sweating, in keeping with a previous study using the LIS in a small sample (n = 13) of DEB patients [14] and in two other studies using a questionnaire [11] or semi-structured interview [15]. Our cohort also associated being stressed out with itching, a factor identified in one study, [11] but less so with changes in weather except in RDEB-S. Our data demonstrated less itch occurrence in a cold environment and on contact with air, consistent with previous reports [11, 14]. Having dry skin has been identified as a factor increasing itch in EB [11, 15] but this is not specifically questioned in the LIS.

The ISA score did not show significant differences between RDEB subtypes or age despite a larger surface area of skin blistering and wounds seen in RDEB-S compared to other subtypes [29]. Although other studies have shown that larger wounds are associated with increased itch, [11, 30] greater self-assessed disease severity, and worse quality of life, [10] itch is prevalent in all types of EB [10] and ISA has only been quantified in one study and not by RDEB subtype [14]. Our results suggest that itch may not be confined to wounds.

Despite high itch frequency, severity and distress in all RDEB subtypes, only 61% of the reviews in our study included medications for itch suggesting a lack of efficacy or satisfaction with these treatments. Emollients and topical corticosteroids reported on medication review but not as itch treatment in the LIS suggests use for other indications (e.g. dry skin, overgranulated wounds). Other studies report similar or an even lower proportion of individuals using medications for itch, with antihistamines being most common [12, 27]. Additionally, patient-reported satisfaction with itch medication was modest. As itch is one of the biggest problems and top priorities for EB patients and caregivers, [15, 28, 31, 32] this suggests that effective itch treatment remains a significant unmet need in EB.

Few clinical trials in EB have evaluated pruritus as a primary or secondary outcome. Studies of mesenchymal stromal cell infusions in RDEB have demonstrated reduced itch as a secondary endpoint [33–35] and a recent randomised, double-blind, placebo-controlled trial of calcipotriol in DEB wounds found a statistically significant fall in itch scores [36]. Another phase 2 randomised, double-blind, placebo-controlled trial of the neurokinin 1 receptor antagonist, serlopitant, included itch reduction as a primary endpoint in different types of EB; although not reaching statistical significance, results showed a trend for greater itch score reduction in the treatment arm [17]. Encouragingly, two clinical trials are underway specifically to address itch in EB; pregabalin in RDEB (NCT 03928093), and topical cannabinol in different types of EB (NCT 04908215).

Different modalities have been used in DEB pruriginosa in individual cases or small series including thalidomide, [37] ciclosporin, [38, 39] intravenous immunoglobulin, [40] topical ketamine and amitriptyline gel with oral mirtazapine [41] and oral janus kinase inhibitors [42, 43]. Recent reports have demonstrated encouraging responses with omalizumab anti-immunoglobulin E monoclonal antibody, [44] and dupilumab against interleukin 4 and interleukin 13 [45–49]. As yet, however, there have been no formal clinical trials in DEB pruriginosa presumably due to its rarity and the inherent difficulties for sufficient statistical powering.

Interestingly, correlation between the iscorEB itch scores and LIS severity and frequency was generally poor across different RDEB subtypes. Similarly, total iscorEB scores showed strong correlations only in RDEB-I for itch frequency, distress and consequences, and RDEB-Inv for itch severity, distress, consequences and ISA. In general, iscorEB total score and LIS subdomains did not correlate in RDEB-S; this may reflect the greater global severity of this subtype whereby the specific contribution made by the different elements of pruritus captured in the LIS are overshadowed by other iscorEB components. Similarly, total QOLEB scores correlated strongly only for RDEB-Inv itch severity, distress, consequences and ISA, but not in RDEB-S suggesting that the contribution of itch to total disease burden captured by QOLEB is minor compared to other facets of the disease. These results suggest that future clinical trials in RDEB where itch is a potential outcome measure should include specific itch measurement tools, such as LIS, rather than relying on more global scoring systems to detect changes.

Limitations of our study include the lack of validation for using LIS under 18 years, the exclusion of children under 8 years who were not asked to complete LIS, and incomplete data for some reviews, especially for ISA. The rarity of RDEB-Pru also meant that the number of participants and reviews was limited which constrained calculations of statistical significance.

## Conclusions

Our study details the specific nature of itch in a large cohort of different RDEB subtypes using the LIS. It demonstrates that individuals with RDEB-S have greater itch frequency, severity and distress than those with RDEB-I, and that RDEB-Pru in particular is associated with high itch severity, duration, frequency, distress and consequences. These differences highlight the limitations of grouping all RDEB patients together when analysing itch. The relatively low frequency of itch medication might indicate lack of efficacy and highlights the need for more effective therapies in EB.

The poor correlation between specific LIS subdomains and disease severity score (iscorEB) and quality of life (QOLEB) for individuals with RDEB-S are counter to previous self-reported studies evaluating itch but may indicate that its impact is dwarfed in these measures by other elements contributing to disease severity.

The data presented here are the largest to date looking specifically at itch in different subtypes of EB and may serve as comparator control data for future clinical trials where the rarity of RDEB makes adequate powering of studies with placebo controls challenging.

### Electronic supplementary material

Below is the link to the electronic supplementary material.

**Additional file 1** Pairwise comparisons between RDEB subtypes by LIS parameter (n = 50). Pairwise comparisons were computed using the Mann-Witney test. Significant differences are indicated in bold. Only the first complete recorded LIS of each participant is considered. As we have not adjusted for multiple comparisons, we would expect two significant results to occur by chance

**Additional file 2** (a) Itch frequency by age group and RDEB subtype (n = 243 from 50 participants). Never: no itch experienced in the preceding month; Rarely: itch 1 to a few times per month; Sometimes: itch 1 to a few times per week; Often: itch 1 to a few times per day; Always: constant itch. (b) Itch duration by age group and RDEB subtype (n = 226 from 48 participants). (c) Itch severity by age group and RDEB subtype (n = 225 from 43 participants)

**Additional file 3** Itch period, circumstances and characteristics by subtype (n = 43). Results are presented as n (%). Only the index review LIS of each participant is considered

**Additional file 4** (a) Itch distress by age group and RDEB subtype (n = 224 from 48 participants). (b) Itch consequences score by age group and RDEB subtype (n = 223 from 48 participants). (c) Itch surface area by age group and RDEB subtype (n = 165 from 47 participants)

**Additional file 5** Itch consequences by subtype (n = 223, from 48 participants). Results presented as n (%)

**Additional file 6** Itch circumstances by subtype (n = 227, from 48 participants). Results presented as n (%)

**Additional file 7** Itch characteristics by subtype (n = 227, from 48 participants). Results presented as n (%)

**Additional file 8** Treatment use by subtype (n = 50). Results are presented as x/n (%), where x is the number of participants reporting use and n is the total number of participants. Only the index review LIS of each participant is considered

**Additional file 9** Satisfaction with itch medication (LIS question 7). Results are scored out of 100 (maximum satisfaction) and are presented as mean (sd) for index and all reviews

**Additional file 10** Correlation between iscorEB itch score and itch frequency and itch severity by subtype. Results are presented as correlation [95% CI] (n) and were calculated using Spearman’s rank correlation. Correlations for sample sizes smaller than 10 should be considered with caution as the associations could be spurious. Correlations could not be calculated for very small sample sizes. Associations are significant if the 95% CI does not contain 0. Correlations can be interpreted as a negligible relationship (< 0.2), weak relationship (0.2–0.4), moderate relationship (0.4–0.6), strong relationship (0.6–0.8), or very strong relationship (> 0.8)

**Additional file 11** Correlation between total iscorEB score and LIS domains by subtype at index review. Results are presented as correlation [95% CI] (n) and were calculated using Spearman’s rank correlation. Correlations for sample sizes smaller than 10 should be considered with caution as the associations could be spurious. Correlations could not be calculated for very small sample sizes. Associations are significant if the 95% CI does not contain 0. Correlations can be interpreted as a negligible relationship (< 0.2), weak relationship (0.2–0.4), moderate relationship (0.4–0.6), strong relationship (0.6–0.8), or very strong relationship (> 0.8)

**Additional file 12** Correlation between total iscorEB score and LIS domains by subtype for all eligible reviews. Results are presented as correlation [95% CI] (n) and were calculated using Spearman’s rank correlation. Correlations for sample sizes smaller than 10 should be considered with caution as the associations could be spurious. Correlations could not be calculated for very small sample sizes. Associations are significant if the 95% CI does not contain 0. Correlations can be interpreted as a negligible relationship (< 0.2), weak relationship (0.2–0.4), moderate relationship (0.4–0.6), strong relationship (0.6–0.8), or very strong relationship (> 0.8)

**Additional file 13** Correlation between total QOLEB score and LIS domains by subtype at index review. Results are presented as correlation [95% CI] (n) and were calculated using Spearman’s rank correlation. Correlations for sample sizes smaller than 10 should be considered with caution as the associations could be spurious. Correlations could not be calculated for very small sample sizes. Associations are significant if the 95% CI does not contain 0. Correlations can be interpreted as a negligible relationship (< 0.2), weak relationship (0.2–0.4), moderate relationship (0.4–0.6), strong relationship (0.6–0.8), or very strong relationship (> 0.8)

**Additional file 14** Correlation between total QOLEB score and LIS domains by subtype for all eligible reviews. Results are presented as correlation [95% CI] (n) and were calculated using Spearman’s rank correlation. Correlations for sample sizes smaller than 10 should be considered with caution as the associations could be spurious. Correlations could not be calculated for very small sample sizes. Associations are significant if the 95% CI does not contain 0. Correlations can be interpreted as a negligible relationship (< 0.2), weak relationship (0.2–0.4), moderate relationship (0.4–0.6), strong relationship (0.6–0.8), or very strong relationship (> 0.8)

## Data Availability

the datasets generated and analysed during the current study are not publicly available as the authors intend to prepare further publications from them. However, the authors would consider reasonable requests to access the data and will make these available in an accessible repository once all relevant data has been published.

## Abbreviations

EB: Epidermolysis bullosa
EBS: Epidermolysis bullosa simplex
DEB: Dystrophic epidermolysis bullosa
ISA: Itch surface area
JEB: Junctional epidermolysis bullosa
KEB: Kindler EB
LIS: Leuven Itch Scale
QOL: Quality of life
RDEB: Recessive dystrophic epidermolysis bullosa
RDEB-I: Recessive dystrophic epidermolysis bullosa intermediate
RDEB-Inv: Recessive dystrophic epidermolysis bullosa inversa
RDEB-Pru: Recessive dystrophic epidermolysis bullosa pruriginosa
RDEB-S: Recessive dystrophic epidermolysis bullosa severe
SCC: Squamous cell carcinoma
