# Supplementary material for: Itch in recessive dystrophic epidermolysis bullosa: findings of PEBLES, a prospective register study
Source: Orphanet J Rare Dis. 2023 Aug 9;18:235. doi: 10.1186/s13023-023-02817-z (PMC10410928; doi:10.1186/s13023-023-02817-z)
Supplement: Supplementary file 1 — Additional file 1 Pairwise comparisons between RDEB subtypes by LIS parameter (n = 50). Pairwise comparisons were computed using the Mann-Witney test. Significant differences are indicated in bold. Only the first complete recorded LIS of each participant is considered. As we have not adjusted for multiple comparisons, we would expect two significant results to occur by chance [file 13023_2023_2817_MOESM1_ESM.docx]

| Subtypes | | LIS parameter | | | | | |
| --- | --- | --- | --- | --- | --- | --- | --- |
| Group 1 | Group 2 | Frequency | Duration | Severity | Distress | Cons | Surface area |
| RDEB-S | RDEB-I | **0.009** | 0.382 | **0.023** | **0.023** | 0.071 | 0.332 |
| RDEB-S | RDEB-Inv | 0.157 | 0.500 | 0.354 | 0.452 | 0.931 | 0.952 |
| RDEB-S | RDEB-Pru | 0.234 | **0.019** | **0.035** | 0.213 | 0.076 | 0.125 |
| RDEB-I | RDEB-Inv | 0.578 | 1.000 | 0.881 | 0.360 | 0.189 | 0.502 |
| RDEB-I | RDEB-Pru | 0.056 | 0.077 | **0.020** | **0.021** | **0.010** | 0.101 |
| RDEB-Inv | RDEB-Pru | 0.149 | 0.120 | 0.067 | 0.117 | 0.105 | 0.253 |

**Additional file 1** Pairwise comparisons between RDEB subtypes by LIS parameter (n=50). Pairwise comparisons were computed using the Mann-Witney test. Significant differences are indicated in bold. Only the first complete recorded LIS of each participant is considered. As we have not adjusted for multiple comparisons, we would expect two significant results to occur by chance.
