# Supplementary material for: Itch in recessive dystrophic epidermolysis bullosa: findings of PEBLES, a prospective register study
Source: Orphanet J Rare Dis. 2023 Aug 9;18:235. doi: 10.1186/s13023-023-02817-z (PMC10410928; doi:10.1186/s13023-023-02817-z)
Supplement: Supplementary file 2 — Additional file 2 (a) Itch frequency by age group and RDEB subtype (n = 243 from 50 participants). Never: no itch experienced in the preceding month; Rarely: itch 1 to a few times per month; Sometimes: itch 1 to a few times per week; Often: itch 1 to a few times per day; Always: constant itch. (b) Itch duration by age group and RDEB subtype (n = 226 from 48 participants). (c) Itch severity by age group and RDEB subtype (n = 225 from 43 participants) [file 13023_2023_2817_MOESM2_ESM.docx]

| Subtype | Overall | RDEB-S | RDEB-I | RDEB-Inv | RDEB-Pru |
| --- | --- | --- | --- | --- | --- |
| n | 43 | 19 | 14 | 7 | 3 |
| *Itch period: when did the itching occur?* | | | | | |
| In the morning | 16 (37) | 5 (26) | 6 (43) | 3 (43) | 2 (67) |
| During the day | 27 (63) | 10 (53) | 9 (64) | 6 (86) | 2 (67) |
| In the evening | 26 (60) | 13 (68) | 8 (57) | 3 (43) | 2 (67) |
| At night | 33 (77) | 16 (84) | 7 (50) | 7 (100) | 3 (100) |
| *Itch circumstances* |  |  |  |  |  |
| During a change in the weather | 19 (44) | 12 (63) | 3 (21) | 3 (43) | 1 (33) |
| During spells of pain | 13 (30) | 5 (26) | 4 (29) | 2 (29) | 2 (67) |
| When making a movement | 14 (33) | 6 (32) | 5 (36) | 1 (14) | 2 (67) |
| When sweating | 20 (47) | 12 (63) | 3 (21) | 3 (43) | 2 (67) |
| In a hot environment | 26 (60) | 15 (79) | 5 (36) | 3 (43) | 3 (100) |
| In a cold environment | 6 (14) | 2 (11) | 3 (21) | 0 (0) | 1 (33) |
| When standing up after sitting or lying down | 9 (21) | 2 (11) | 5 (36) | 0 (0) | 2 (67) |
| When I was stressed out | 23 (53) | 14 (74) | 4 (29) | 3 (43) | 2 (67) |
| On contact with air | 8 (19) | 5 (26) | 0 (0) | 1 (14) | 2 (67) |
| When touching the skin | 13 (30) | 5 (26) | 4 (29) | 2 (29) | 2 (67) |
| *Itch characteristics: how did the itching manifest itself?* | | | | | |
| A tickling sensation | 29 (67) | 13 (68) | 10 (71) | 4 (57) | 2 (67) |
| A tingling sensation | 8 (19) | 6 (32) | 0 (0) | 0 (0) | 2 (67) |
| A prickling sensation | 19 (44) | 7 (37) | 6 (43) | 4 (57) | 2 (67) |
| A stinging sensation | 6 (14) | 3 (16) | 1 (7) | 1 (14) | 1 (33) |
| A burning sensation | 12 (28) | 6 (32) | 2 (14) | 2 (29) | 2 (67) |

**Additional file 3** Itch period, circumstances and characteristics by subtype (n=43). Results are presented as n (%). Only the index review LIS of each participant is considered.
