# Supplementary material for: Itch in recessive dystrophic epidermolysis bullosa: findings of PEBLES, a prospective register study
Source: Orphanet J Rare Dis. 2023 Aug 9;18:235. doi: 10.1186/s13023-023-02817-z (PMC10410928; doi:10.1186/s13023-023-02817-z)
Supplement: Supplementary file 3 — Additional file 3 Itch period, circumstances and characteristics by subtype (n = 43). Results are presented as n (%). Only the index review LIS of each participant is considered [file 13023_2023_2817_MOESM3_ESM.docx]

| Consequences of itching? | Subtype | Never | Rarely | Sometimes | Often | Always |
| --- | --- | --- | --- | --- | --- | --- |
| Lesions from scratching | RDEB-S | 7 (7) | 15 (15) | 40 (40) | 22 (22) | 15 (15) |
|  | RDEB-I | 12 (16) | 10 (13) | 27 (36) | 12 (16) | 14 (19) |
|  | RDEB-Inv | 3 (8) | 6 (15) | 10 (26) | 9 (23) | 11 (28) |
|  | RDEB-Pru | 0 (0) | 0 (0) | 2 (20) | 6 (60) | 2 (20) |
| Reduced social contact due to itching | RDEB-S | 67 (68) | 21 (21) | 7 (7) | 3 (3) | 1 (1) |
|  | RDEB-I | 54 (72) | 14 (19) | 3 (4) | 2 (3) | 2 (3) |
|  | RDEB-Inv | 19 (49) | 8 (21) | 9 (23) | 3 (8) | - |
|  | RDEB-Pru | 2 (20) | 3 (30) | 4 (40) | 1 (10) | - |
| Reduced quality of life due to itching | RDEB-S | 31 (31) | 27 (27) | 26 (26) | 10 (10) | 5 (5) |
|  | RDEB-I | 46 (61) | 12 (16) | 8 (11) | 7 (9) | 2 (3) |
|  | RDEB-Inv | 16 (41) | 9 (23) | 6 (15) | 8 (21) | - |
|  | RDEB-Pru | 1 (10) | 0 (0) | 0 (0) | 4 (40) | 5 (50) |
| Disturbed my routine activities due to itching | RDEB-S | 20 (20) | 22 (22) | 38 (38) | 13 (13) | 6 (6) |
|  | RDEB-I | 43 (57) | 12 (16) | 12 (16) | 6 (8) | 2 (3) |
|  | RDEB-Inv | 12 (31) | 14 (36) | 6 (15) | 7 (18) | - |
|  | RDEB-Pru | 0 (0) | 0 (0) | 2 (20) | 6 (60) | 2 (20) |
| Difficulties in falling asleep due to itching | RDEB-S | 12 (12) | 13 (13) | 38 (38) | 24 (24) | 12 (12) |
|  | RDEB-I | 29 (39) | 10 (13) | 19 (25) | 12 (16) | 5 (7) |
|  | RDEB-Inv | 8 (21) | 7 (18) | 11 (28) | 8 (21) | 5 (13) |
|  | RDEB-Pru | 0 (0) | 1 (10) | 1 (10) | 4 (40) | 4 (40) |
| Waking up due to itching | RDEB-S | 23 (23) | 23 (23) | 31 (31) | 11 (11) | 11 (11) |
|  | RDEB-I | 33 (44) | 11 (15) | 15 (20) | 11 (15) | 5 (7) |
|  | RDEB-Inv | 4 (10) | 11 (28) | 10 (26) | 10 (26) | 4 (10) |
|  | RDEB-Pru | 0 (0) | 0 (0) | 1 (10) | 6 (60) | 3 (30) |
| Needed sleeping pills due to itching | RDEB-S | 79 (80) | 4 (4) | 5 (5) | 4 (4) | 7 (7) |
|  | RDEB-I | 72 (96) | 1 (1) | 1 (1) | 1 (1) | - |
|  | RDEB-Inv | 26 (67) | 4 (10) | 2 (5) | 5 (13) | 2 (5) |
|  | RDEB-Pru | 2 (20) | - | 1 (10) | 2 (20) | 5 (50) |
| Loss of appetite due to itching | RDEB-S | 76 (77) | 12 (12) | 8 (8) | 1 (1) | 2 (2) |
|  | RDEB-I | 62 (83) | 5 (7) | 4 (5) | 3 (4) | 1 (1) |
|  | RDEB-Inv | 27 (69) | 7 (18) | 4 (10) | 1 (3) | - |
|  | RDEB-Pru | 6 (60) | 2 (20) | 2 (20) | - | - |
| Bad mood due to itching | RDEB-S | 18 (18) | 17 (17) | 39 (39) | 20 (20) | 5 (5) |
|  | RDEB-I | 41 (55) | 10 (13) | 14 (19) | 7 (9) | 3 (4) |
|  | RDEB-Inv | 12 (31) | 8 (21) | 11 (28) | 8 (21) | - |
|  | RDEB-Pru | 0 (0) | 2 (20) | 2 (20) | 3 (30) | 3 (30) |
| Changes in behavior toward others due to itching | RDEB-S | 32 (32) | 16 (16) | 36 (36) | 12 (12) | 3 (3) |
|  | RDEB-I | 61 (81) | 7 (9) | 5 (7) | 2 (3) | - |
|  | RDEB-Inv | 20 (51) | 6 (15) | 10 (26) | 3 (8) | - |
|  | RDEB-Pru | 2 (20) | 2 (20) | 3 (30) | 2 (20) | 1 (10) |
| Loss of concentration due to itching | RDEB-S | 12 (12) | 20 (20) | 44 (44) | 19 (19) | 4 (4) |
|  | RDEB-I | 38 (51) | 12 (16) | 20 (27) | 5 (7) | - |
|  | RDEB-Inv | 14 (36) | 8 (21) | 10 (26) | 7 (18) | - |
|  | RDEB-Pru | 0 (0) | 0 (0) | 1 (10) | 7 (70) | 2 (20) |

**Additional file 5** Itch consequences by subtype (n = 223, from 48 participants). Results presented as n (%).
