# Supplementary material for: Itch in recessive dystrophic epidermolysis bullosa: findings of PEBLES, a prospective register study
Source: Orphanet J Rare Dis. 2023 Aug 9;18:235. doi: 10.1186/s13023-023-02817-z (PMC10410928; doi:10.1186/s13023-023-02817-z)
Supplement: Supplementary file 4 — Additional file 4 (a) Itch distress by age group and RDEB subtype (n = 224 from 48 participants). (b) Itch consequences score by age group and RDEB subtype (n = 223 from 48 participants). (c) Itch surface area by age group and RDEB subtype (n = 165 from 47 participants) [file 13023_2023_2817_MOESM4_ESM.docx]

|  | Subtype | | | | |
| --- | --- | --- | --- | --- | --- |
|  | RDEB-S | RDEB-I | RDEB-Inv | RDEB-Pru | Overall |
| iscorEB itch score vs LIS itch frequency | 0.16 [-0.04,0.35] (n = 101) | 0.50 [0.32,0.64] (n = 85) | 0.36 [0.08,0.59] (n = 46) | 0.07 [-0.58,0.67] (n = 10) | 0.36 [0.25,0.47] (n = 242) |
| iscorEB itch score vs LIS itch severity | 0.13 [-0.07,0.32] (n = 99) | 0.45 [0.25,0.62] (n = 76) | 0.32 [0.01,0.58] (n = 39) | 0.15 [-0.53,0.71] (n = 10) | 0.33 [0.21,0.44] (n = 224) |

**Additional file 10** Correlation between iscorEB itch score and itch frequency and itch severity by subtype. Results are presented as correlation [95% CI] (n) and were calculated using Spearman’s rank correlation. Correlations for sample sizes smaller than 10 should be considered with caution as the associations could be spurious. Correlations could not be calculated for very small sample sizes. Associations are significant if the 95% CI does not contain 0. Correlations can be interpreted as a negligible relationship (<0.2), weak relationship (0.2-0.4), moderate relationship (0.4-0.6), strong relationship (0.6-0.8), or very strong relationship (>0.8).
