# Supplementary material for: Itch in recessive dystrophic epidermolysis bullosa: findings of PEBLES, a prospective register study
Source: Orphanet J Rare Dis. 2023 Aug 9;18:235. doi: 10.1186/s13023-023-02817-z (PMC10410928; doi:10.1186/s13023-023-02817-z)
Supplement: Supplementary file 5 — Additional file 5 Itch consequences by subtype (n = 223, from 48 participants). Results presented as n (%) [file 13023_2023_2817_MOESM5_ESM.docx]

|  | Subtype | | | | |
| --- | --- | --- | --- | --- | --- |
|  | RDEB-S | RDEB-I | RDEB-Inv | RDEB-Pru | Overall |
| Total iscorEB score vs LIS itch frequency | 0.04 [-0.46,0.53] (n = 16) | 0.65 [0.18,0.88] (n = 14) | 0.55 [-0.18,0.89] (n = 9) | n/a (n = 3) | 0.62 [0.39,0.78] (n = 42) |
| Total iscorEB score vs LIS itch duration | 0.47 [-0.03,0.78] (n = 16) | 0.36 [-0.27,0.78] (n = 12) | 0.81 [0.14,0.97] (n = 7) | n/a (n = 3) | 0.34 [0.02,0.59] (n = 38) |
| Total iscorEB score vs LIS itch severity | -0.27 [-0.68,0.26] (n = 16) | 0.61 [0.06,0.88] (n = 12) | 0.89 [0.43,0.98] (n = 7) | n/a (n = 3) | 0.48 [0.18,0.69] (n = 38) |
| Total iscorEB score vs LIS itch distress | -0.32 [-0.71,0.21] (n = 16) | 0.33 [-0.30,0.76] (n = 12) | 0.71 [-0.08,0.95] (n = 7) | n/a (n = 3) | 0.24 [-0.09,0.52] (n = 38) |
| Total iscorEB score vs LIS itch consequences | 0.30 [-0.23,0.69] (n = 16) | 0.60 [0.04,0.87] (n = 12) | 0.95 [0.67,0.99] (n = 7) | n/a (n = 3) | 0.49 [0.20,0.70] (n = 38) |
| Total iscorEB score vs LIS itch surface area | 0.35 [-0.20,0.73] (n = 15) | 0.42 [-0.20,0.80] (n = 12) | 0.77 [0.05,0.96] (n = 7) | n/a (n = 3) | 0.41 [0.10,0.65] (n = 37) |

**Additional file 11** Correlation between total iscorEB score and LIS domains by subtype at index review. Results are presented as correlation [95% CI] (n) and were calculated using Spearman’s rank correlation. Correlations for sample sizes smaller than 10 should be considered with caution as the associations could be spurious. Correlations could not be calculated for very small sample sizes. Associations are significant if the 95% CI does not contain 0. Correlations can be interpreted as a negligible relationship (<0.2), weak relationship (0.2-0.4), moderate relationship (0.4-0.6), strong relationship (0.6-0.8), or very strong relationship (>0.8).
