# Supplementary material for: Itch in recessive dystrophic epidermolysis bullosa: findings of PEBLES, a prospective register study
Source: Orphanet J Rare Dis. 2023 Aug 9;18:235. doi: 10.1186/s13023-023-02817-z (PMC10410928; doi:10.1186/s13023-023-02817-z)
Supplement: Supplementary file 7 — Additional file 7 Itch characteristics by subtype (n = 227, from 48 participants). Results presented as n (%) [file 13023_2023_2817_MOESM7_ESM.docx]

|  | Subtype | | | | |
| --- | --- | --- | --- | --- | --- |
|  | RDEB-S | RDEB-I | RDEB-Inv | RDEB-Pru | Overall |
| Total QOLEB score vs LIS itch frequency | 0.11 [-0.47,0.63] (n = 13) | 0.58 [0.14,0.83] (n = 17) | 0.45 [-0.31,0.86] (n = 9) | n/a (n = 3) | 0.64 [0.41,0.79] (n = 42) |
| Total QOLEB itch score vs LIS itch duration | 0.50 [-0.06,0.83] (n = 13) | 0.52 [-0.04,0.83] (n = 13) | 0.81 [0.14,0.97] (n = 7) | n/a (n = 3) | 0.47 [0.17,0.69] (n = 36) |
| Total QOLEB itch score vs LIS itch severity | -0.28 [-0.72,0.32] (n = 13) | 0.08 [-0.49,0.60] (n = 13) | 0.89 [0.43,0.98] (n = 7) | n/a (n = 3) | 0.42 [0.10,0.66] (n = 36) |
| Total QOLEB itch score vs LIS itch distress | -0.04 [-0.58,0.52] (n = 13) | 0.25 [-0.35,0.70] (n = 13) | 0.71 [-0.08,0.95] (n = 7) | n/a (n = 3) | 0.42 [0.10,0.66] (n = 36) |
| Total QOLEB itch score vs LIS itch consequences | 0.28 [-0.32,0.72] (n = 13) | 0.57 [0.02,0.85] (n = 13) | 0.95 [0.67,0.99] (n = 7) | n/a (n = 3) | 0.56 [0.29,0.75] (n = 36) |
| Total QOLEB itch score vs LIS itch surface area | 0.64 [0.11,0.89] (n = 12) | 0.42 [-0.17,0.79] (n = 13) | 0.77 [0.05,0.96] (n = 7) | n/a (n = 3) | 0.51 [0.22,0.72] (n = 35) |

**Additional file 13** Correlation between total QOLEB score and LIS domains by subtype at index review. Results are presented as correlation [95% CI] (n) and were calculated using Spearman’s rank correlation. Correlations for sample sizes smaller than 10 should be considered with caution as the associations could be spurious. Correlations could not be calculated for very small sample sizes. Associations are significant if the 95% CI does not contain 0. Correlations can be interpreted as a negligible relationship (<0.2), weak relationship (0.2-0.4), moderate relationship (0.4-0.6), strong relationship (0.6-0.8), or very strong relationship (>0.8).
