# Supplementary material for: Itch in recessive dystrophic epidermolysis bullosa: findings of PEBLES, a prospective register study
Source: Orphanet J Rare Dis. 2023 Aug 9;18:235. doi: 10.1186/s13023-023-02817-z (PMC10410928; doi:10.1186/s13023-023-02817-z)
Supplement: Supplementary file 8 — Additional file 8 Treatment use by subtype (n = 50). Results are presented as x/n (%), where x is the number of participants reporting use and n is the total number of participants. Only the index review LIS of each participant is considered [file 13023_2023_2817_MOESM8_ESM.docx]

|  | Subtype | | | | |
| --- | --- | --- | --- | --- | --- |
|  | RDEB-S | RDEB-I | RDEB-Inv | RDEB-Pru | Overall |
| Total QOLEB score vs LIS itch frequency | 0.18 [-0.06,0.40] (n = 71) | 0.55 [0.38,0.69] (n = 81) | 0.58 [0.34,0.75] (n = 44) | 0.07 [-0.58,0.67] (n = 10) | 0.58 [0.48,0.66] (n = 206) |
| Total QOLEB itch score vs LIS itch duration | 0.39 [0.17,0.57] (n = 71) | 0.32 [0.09,0.51] (n = 72) | 0.41 [0.09,0.64] (n = 37) | 0.35 [-0.36,0.80] (n = 10) | 0.35 [0.22,0.47] (n = 190) |
| Total QOLEB itch score vs LIS itch severity | 0.11 [-0.12,0.34] (n = 70) | 0.34 [0.12,0.53] (n = 72) | 0.83 [0.69,0.91] (n = 37) | 0.38 [-0.33,0.81] (n = 10) | 0.52 [0.41,0.62] (n = 189) |
| Total QOLEB itch score vs LIS itch distress | 0.01 [-0.22,0.25] (n = 70) | 0.50 [0.31,0.66] (n = 72) | 0.76 [0.58,0.87] (n = 38) | 0.63 [-0.01,0.90] (n = 10) | 0.53 [0.41,0.62] (n = 190) |
| Total QOLEB itch score vs LIS itch consequences | 0.30 [0.07,0.50] (n = 70) | 0.60 [0.43,0.73] (n = 71) | 0.87 [0.76,0.93] (n = 37) | 0.39 [-0.31,0.82] (n = 10) | 0.63 [0.54,0.71] (n = 188) |
| Total QOLEB itch score vs LIS itch surface area | 0.41 [0.13,0.62] (n = 46) | 0.45 [0.22,0.64] (n = 58) | 0.65 [0.40,0.81] (n = 33) | 0.41 [-0.60,0.92] (n = 6) | 0.53 [0.40,0.64] (n = 143) |

**Additional file 14** Correlation between total QOLEB score and LIS domains by subtype for all eligible reviews. Results are presented as correlation [95% CI] (n) and were calculated using Spearman’s rank correlation. Correlations for sample sizes smaller than 10 should be considered with caution as the associations could be spurious. Correlations could not be calculated for very small sample sizes. Associations are significant if the 95% CI does not contain 0. Correlations can be interpreted as a negligible relationship (<0.2), weak relationship (0.2-0.4), moderate relationship (0.4-0.6), strong relationship (0.6-0.8), or very strong relationship (>0.8).
