# Supplementary material for: Itch in recessive dystrophic epidermolysis bullosa: findings of PEBLES, a prospective register study
Source: Orphanet J Rare Dis. 2023 Aug 9;18:235. doi: 10.1186/s13023-023-02817-z (PMC10410928; doi:10.1186/s13023-023-02817-z)
Supplement: Supplementary file 9 — Additional file 9 Satisfaction with itch medication (LIS question 7). Results are scored out of 100 (maximum satisfaction) and are presented as mean (sd) for index and all reviews [file 13023_2023_2817_MOESM9_ESM.docx]

|  | | Subtype | | | |
| --- | --- | --- | --- | --- | --- |
| Circumstances | Overall | RDEB-S | RDEB-I | RDEB-Inv | RDEB-Pru |
| n | 227 | 101 | 76 | 40 | 10 |
| During a change in the weather | 99 (44) | 57 (56) | 16 (21) | 19 (48) | 7 (70) |
| During spells of pain | 74 (33) | 35 (35) | 12 (16) | 19 (48) | 8 (80) |
| When making a movement | 59 (26) | 30 (30) | 15 (20) | 8 (20) | 6 (60) |
| When sweating | 118 (52) | 69 (68) | 21 (28) | 20 (50) | 8 (80) |
| In a hot environment | 147 (65) | 84 (83) | 30 (39) | 24 (60) | 9 (90) |
| In a cold environment | 37 (16) | 15 (15) | 11 (14) | 6 (15) | 5 (50) |
| When standing up after sitting or lying down | 49 (22) | 16 (16) | 17 (22) | 10 (25) | 6 (60) |
| When I was stressed out | 128 (56) | 77 (76) | 22 (29) | 22 (55) | 7 (70) |
| On contact with air | 71 (31) | 40 (40) | 14 (18) | 11 (28) | 6 (60) |
| When touching the skin | 79 (35) | 27 (27) | 32 (42) | 14 (35) | 6 (60) |

**Additional file 6** Itch circumstances by subtype (n = 227, from 48 participants). Results presented as n (%).
