# Supplementary material for: Itch in recessive dystrophic epidermolysis bullosa: findings of PEBLES, a prospective register study
Source: Orphanet J Rare Dis. 2023 Aug 9;18:235. doi: 10.1186/s13023-023-02817-z (PMC10410928; doi:10.1186/s13023-023-02817-z)
Supplement: Supplementary file 10 — Additional file 10 Correlation between iscorEB itch score and itch frequency and itch severity by subtype. Results are presented as correlation [95% CI] (n) and were calculated using Spearman’s rank correlation. Correlations for sample sizes smaller than 10 should be considered with caution as the associations could be spurious. Correlations could not be calculated for very small sample sizes. Associations are significant if the 95% CI does not contain 0. Correlations can be interpreted as a negligible relationship (< 0.2), weak relationship (0.2–0.4), moderate relationship (0.4–0.6), strong relationship (0.6–0.8), or very strong relationship (> 0.8) [file 13023_2023_2817_MOESM10_ESM.docx]

|  | | Subtype | | | |
| --- | --- | --- | --- | --- | --- |
| How did the itching manifest itself? | Overall | RDEB-S | RDEB-I | RDEB-Inv | RDEB-Pru |
| n | 227 | 101 | 76 | 40 | 10 |
| A tickling sensation | 139 (61) | 58 (57) | 55 (72) | 19 (48) | 7 (70) |
| A tingling sensation | 69 (30) | 41 (41) | 16 (21) | 4 (10) | 8 (80) |
| A prickling sensation | 105 (46) | 53 (52) | 27 (36) | 19 (48) | 6 (60) |
| A stinging sensation | 42 (19) | 18 (18) | 7 (9) | 13 (32) | 4 (40) |
| A burning sensation | 72 (32) | 39 (39) | 13 (17) | 13 (32) | 7 (70) |

**Additional file 7** Itch characteristics by subtype (n = 227, from 48 participants). Results presented as n (%).
