# Supplementary material for: Itch in recessive dystrophic epidermolysis bullosa: findings of PEBLES, a prospective register study
Source: Orphanet J Rare Dis. 2023 Aug 9;18:235. doi: 10.1186/s13023-023-02817-z (PMC10410928; doi:10.1186/s13023-023-02817-z)
Supplement: Supplementary file 11 — Additional file 11 Correlation between total iscorEB score and LIS domains by subtype at index review. Results are presented as correlation [95% CI] (n) and were calculated using Spearman’s rank correlation. Correlations for sample sizes smaller than 10 should be considered with caution as the associations could be spurious. Correlations could not be calculated for very small sample sizes. Associations are significant if the 95% CI does not contain 0. Correlations can be interpreted as a negligible relationship (< 0.2), weak relationship (0.2–0.4), moderate relationship (0.4–0.6), strong relationship (0.6–0.8), or very strong relationship (> 0.8) [file 13023_2023_2817_MOESM11_ESM.docx]

| Treatment | Overall | RDEB-S | RDEB-I | RDEB-Inv | RDEB-Pru |
| --- | --- | --- | --- | --- | --- |
| Emollient (with or without menthol) | 13/50  (26) | 4/20  (20) | 4/18  (22) | 3/9  (33) | 2/3  (67) |
| Emollient (with or without menthol) only | 11/13  (85) | 3/4  (75) | 4/4  (100) | 3/3  (100) | 1/2  (50) |
| Emollient without menthol | 12/50  (24) | 4/20  (20) | 3/18  (17) | 3/9  (33) | 2/3  (67) |
| Emollient without menthol only | 9/12  (75) | 3/4  (75) | 3/3  (100) | 2/3  (67) | 1/2  (50) |
| Emollient with menthol | 2/50  (4) | 0/20  (0) | 1/18  (6) | 1/9  (11) | 0/3  (0) |
| Emollient with menthol only | 1/2  (50) |  | 1/1  (100) | 0/1  (0) |  |
| Corticosteroid | 1/50  (2) | 1/20  (5) | 0/18  (0) | 0/9  (0) | 0/3  (0) |
| Corticosteroid only | 0/1  (0) | 0/1  (0) |  |  |  |
| Emollient and corticosteroid | 0/50  (0) | 0/20  (0) | 0/18  (0) | 0/9  (0) | 0/3  (0) |
| Antihistamine | 14/50  (28) | 9/20  (45) | 2/18  (11) | 1/9  (11) | 2/3  (67) |
| Antihistamine only | 11/14  (79) | 7/9  (78) | 2/2  (100) | 1/1  (100) | 1/2  (50) |
| Antihistamines (2+) | 7/50  (14) | 5/20  (25) | 0/18  (0) | 1/9  (11) | 1/3  (33) |
| Antihistamine and emollient | 2/50  (4) | 1/20  (5) | 0/18  (0) | 0/9  (0) | 1/3  (33) |
| Antihistamine and corticosteroid | 1/50  (2) | 1/20  (5) | 0/18  (0) | 0/9  (0) | 0/3  (0) |
| Antihistamines, emollients and corticosteroids | 0/50  (0) | 0/20  (0) | 0/18  (0) | 0/9  (0) | 0/3  (0) |
| Antihistamines and emollients or corticosteroids | 3/50  (6) | 2/20  (10) | 0/18  (0) | 0/9  (0) | 1/3  (33) |

**Additional file 8** Treatment use by subtype (n = 50). Results are presented as x/n (%), where x is the number of participants reporting use and n is the total number of participants. Only the index review LIS of each participant is considered.
