# Supplementary material for: Itch in recessive dystrophic epidermolysis bullosa: findings of PEBLES, a prospective register study
Source: Orphanet J Rare Dis. 2023 Aug 9;18:235. doi: 10.1186/s13023-023-02817-z (PMC10410928; doi:10.1186/s13023-023-02817-z)
Supplement: Supplementary file 12 — Additional file 12 Correlation between total iscorEB score and LIS domains by subtype for all eligible reviews. Results are presented as correlation [95% CI] (n) and were calculated using Spearman’s rank correlation. Correlations for sample sizes smaller than 10 should be considered with caution as the associations could be spurious. Correlations could not be calculated for very small sample sizes. Associations are significant if the 95% CI does not contain 0. Correlations can be interpreted as a negligible relationship (< 0.2), weak relationship (0.2–0.4), moderate relationship (0.4–0.6), strong relationship (0.6–0.8), or very strong relationship (> 0.8) [file 13023_2023_2817_MOESM12_ESM.docx]

a


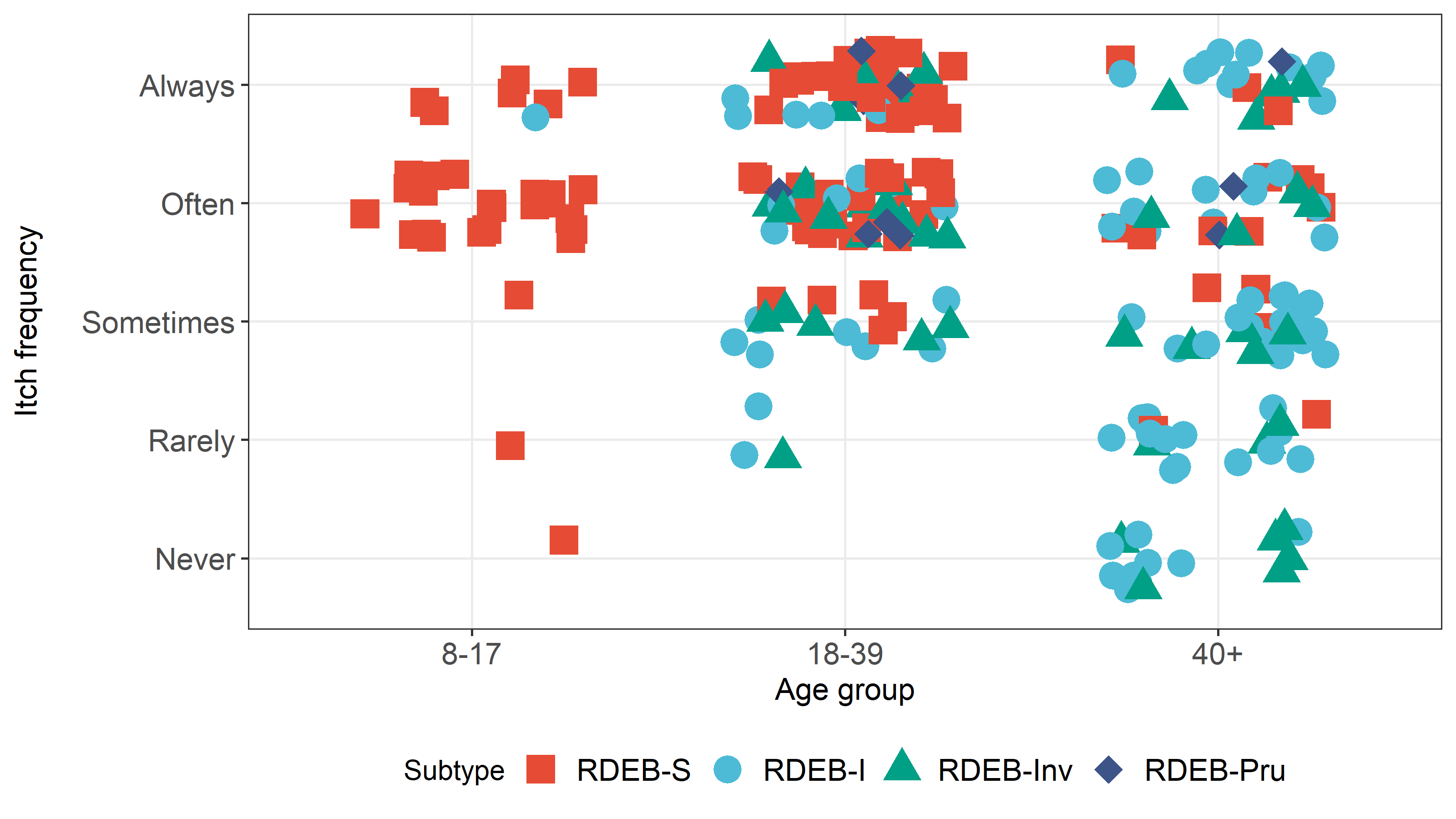


b


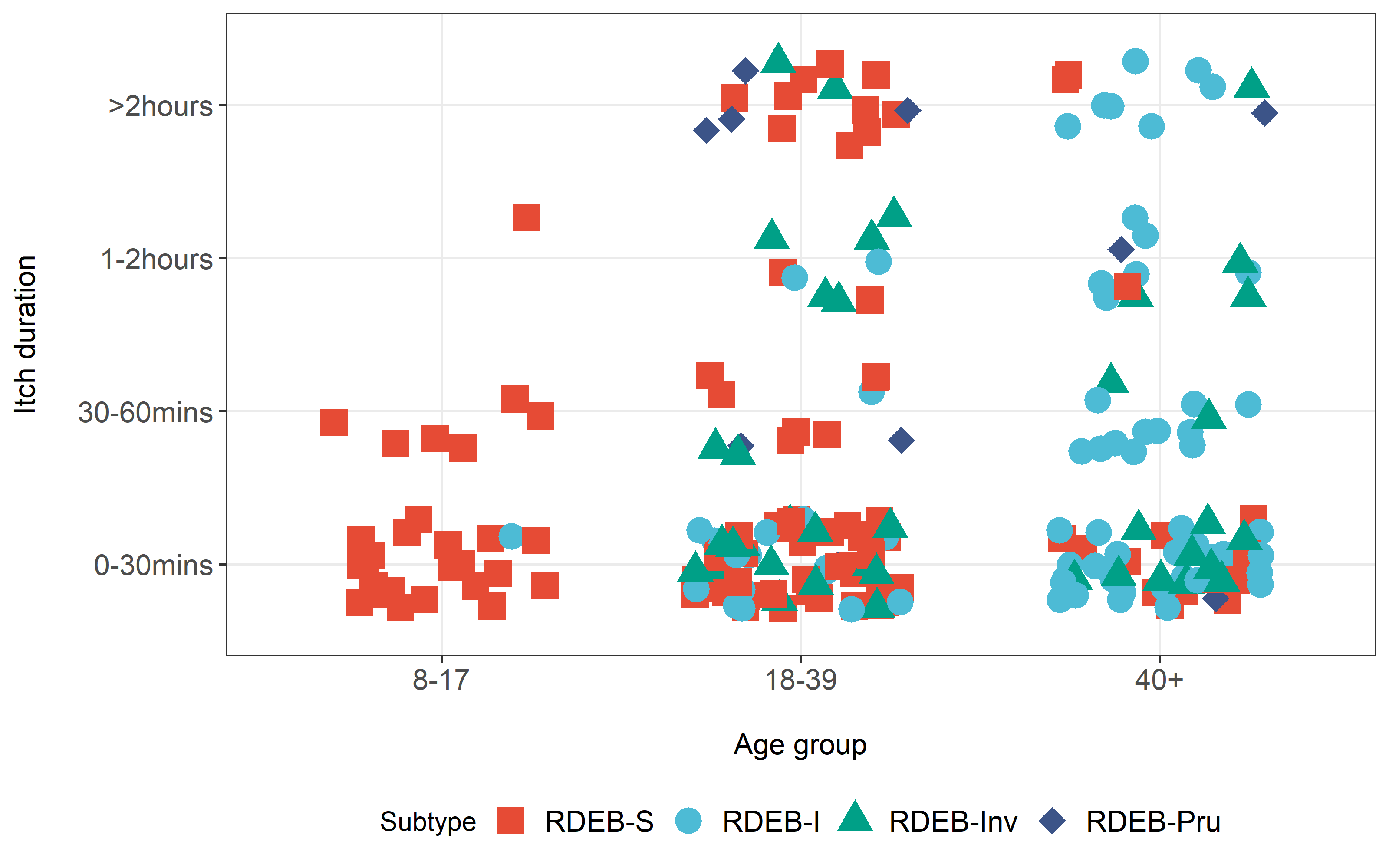


c


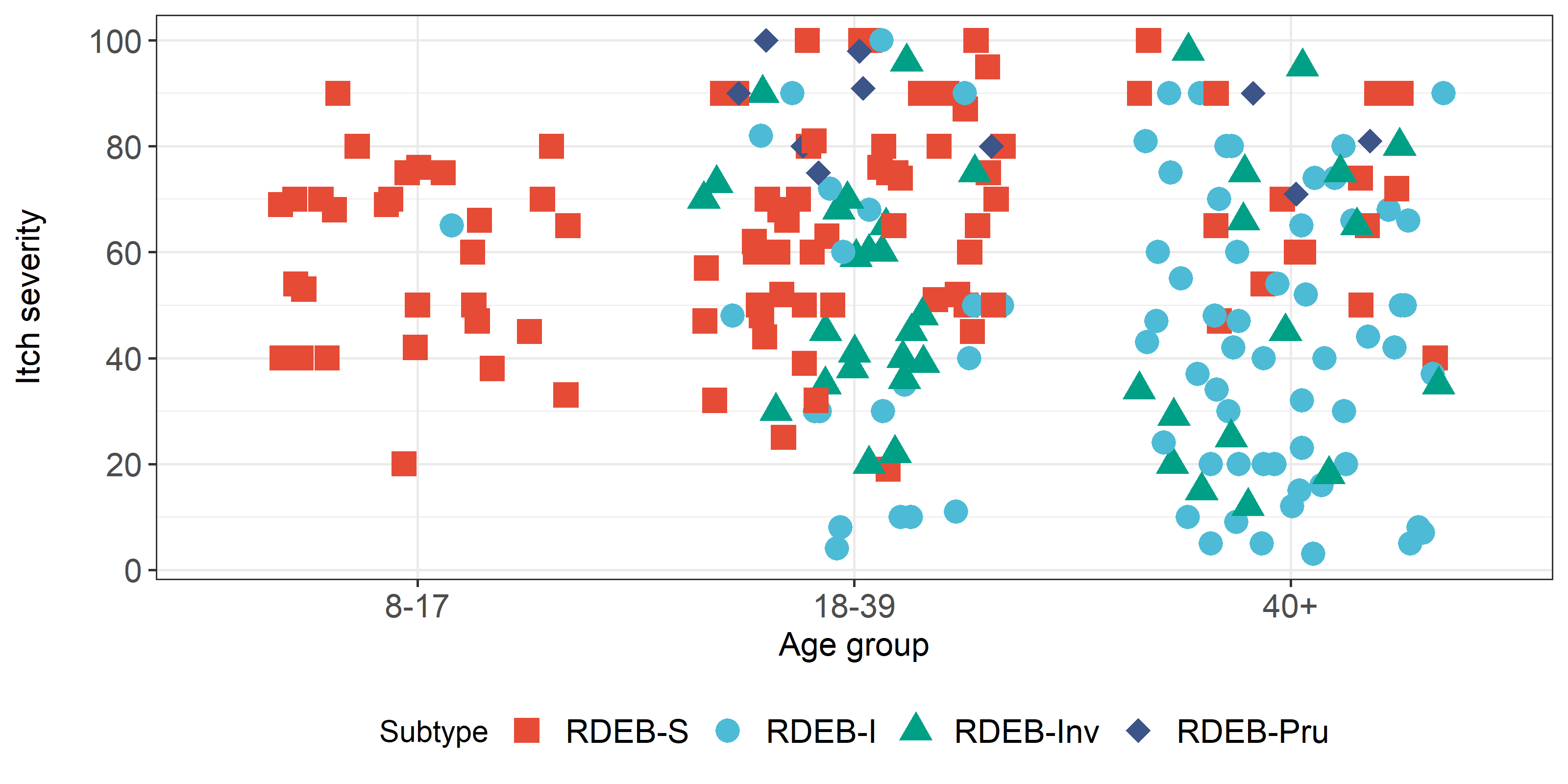


**Additional file 2 (a)** Itch frequency by age group and RDEB subtype (n=243 from 50 participants). Never: no itch experienced in the preceding month; Rarely: itch 1 to a few times per month; Sometimes: itch 1 to a few times per week; Often: itch 1 to a few times per day; Always: constant itch. **(b)** Itch duration by age group and RDEB subtype (n=226 from 48 participants). **(c)** Itch severity by age group and RDEB subtype (n=225 from 43 participants).
