# Supplementary material for: Itch in recessive dystrophic epidermolysis bullosa: findings of PEBLES, a prospective register study
Source: Orphanet J Rare Dis. 2023 Aug 9;18:235. doi: 10.1186/s13023-023-02817-z (PMC10410928; doi:10.1186/s13023-023-02817-z)
Supplement: Supplementary file 13 — Additional file 13 Correlation between total QOLEB score and LIS domains by subtype at index review. Results are presented as correlation [95% CI] (n) and were calculated using Spearman’s rank correlation. Correlations for sample sizes smaller than 10 should be considered with caution as the associations could be spurious. Correlations could not be calculated for very small sample sizes. Associations are significant if the 95% CI does not contain 0. Correlations can be interpreted as a negligible relationship (< 0.2), weak relationship (0.2–0.4), moderate relationship (0.4–0.6), strong relationship (0.6–0.8), or very strong relationship (> 0.8) [file 13023_2023_2817_MOESM13_ESM.docx]

a


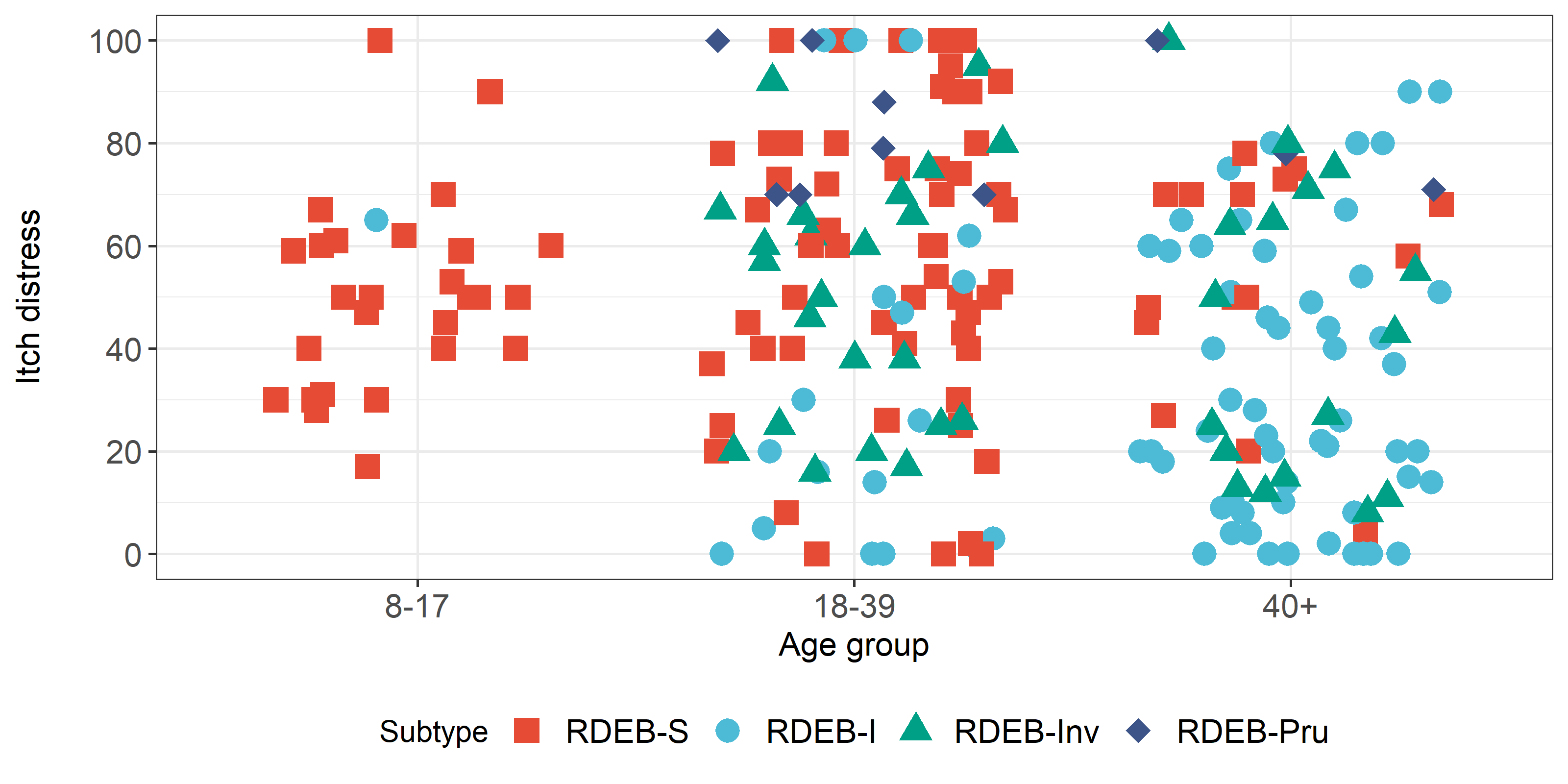


b


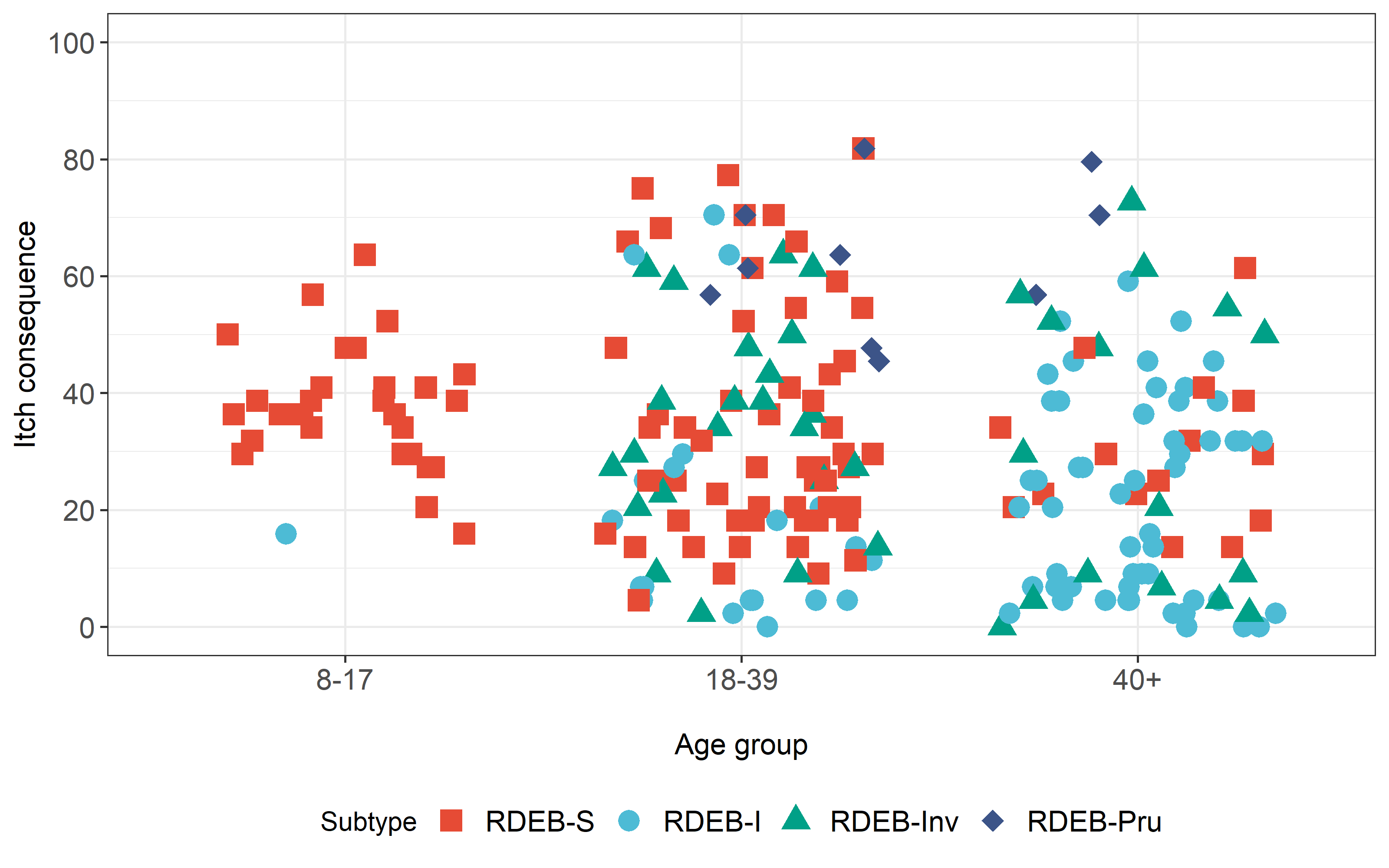


c


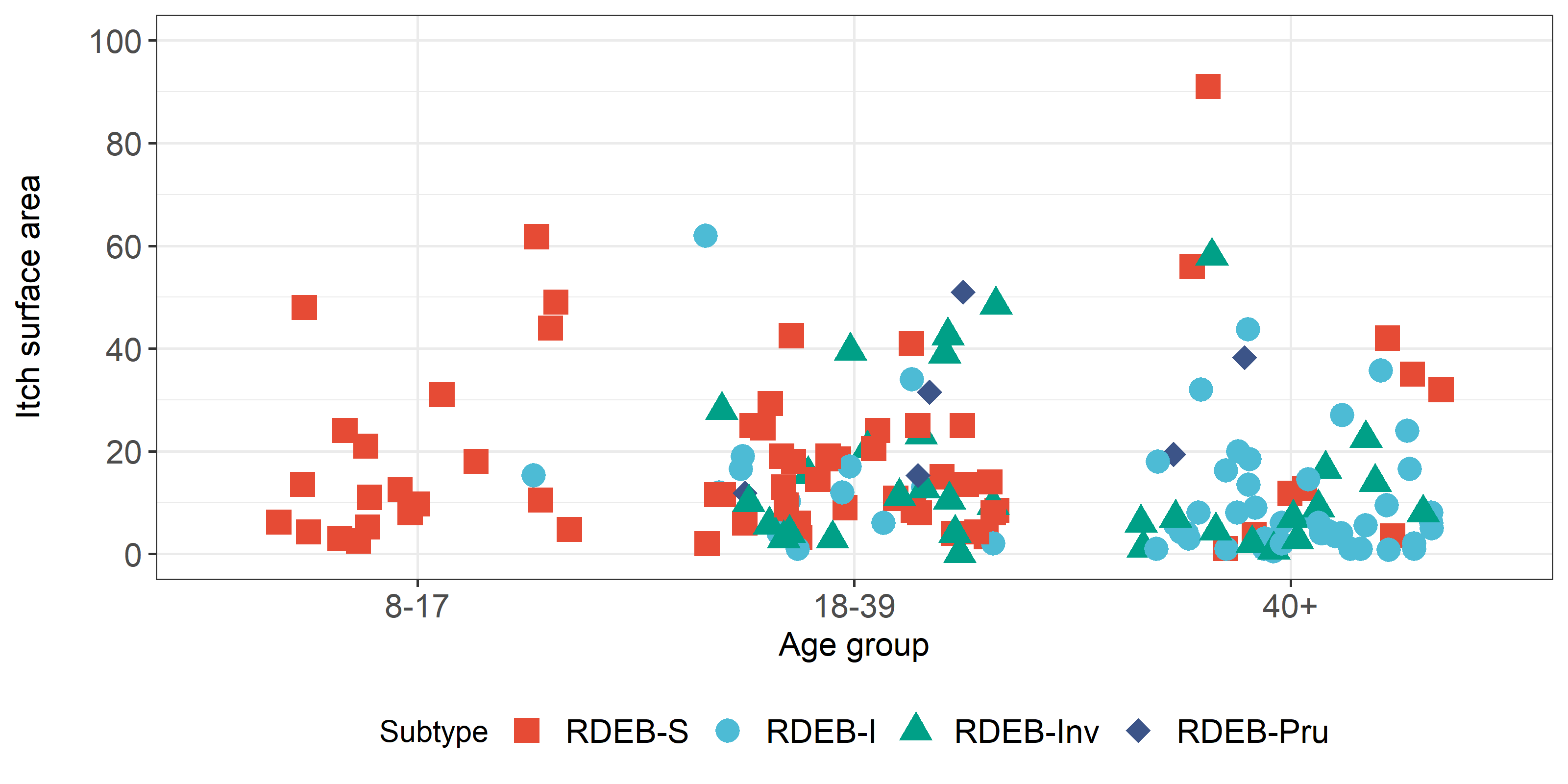


**Additional file 4 (a)** Itch distress by age group and RDEB subtype (n=224 from 48 participants). **(b)** Itch consequences score by age group and RDEB subtype (n=223 from 48 participants). (**c)** Itch surface area by age group and RDEB subtype (n=165 from 47 participants).
