# Supplementary material for: Itch in recessive dystrophic epidermolysis bullosa: findings of PEBLES, a prospective register study
Source: Orphanet J Rare Dis. 2023 Aug 9;18:235. doi: 10.1186/s13023-023-02817-z (PMC10410928; doi:10.1186/s13023-023-02817-z)
Supplement: Supplementary file 14 — Additional file 14 Correlation between total QOLEB score and LIS domains by subtype for all eligible reviews. Results are presented as correlation [95% CI] (n) and were calculated using Spearman’s rank correlation. Correlations for sample sizes smaller than 10 should be considered with caution as the associations could be spurious. Correlations could not be calculated for very small sample sizes. Associations are significant if the 95% CI does not contain 0. Correlations can be interpreted as a negligible relationship (< 0.2), weak relationship (0.2–0.4), moderate relationship (0.4–0.6), strong relationship (0.6–0.8), or very strong relationship (> 0.8) [file 13023_2023_2817_MOESM14_ESM.docx]

|  | Subtype | | | | |
| --- | --- | --- | --- | --- | --- |
|  | RDEB-S | RDEB-I | RDEB-Inv | RDEB-Pru | Overall |
| Satisfaction with itch medication (index review) | 46.0 (29.0) (n = 13) | 42.4 (45.4) (n = 5) | 29.0 (28.6) (n = 4) | 35.7 (28.4) (n = 3) | 41.3 (31.3) (n = 25) |
| Satisfaction with itch medication (all reviews) | 47.0 (29.6) (n = 68) | 58.6 (36.2) (n = 33) | 48.4 (34.1) (n = 22) | 47.8 (28.4) (n = 10) | 50.2 (32.0) (n = 133) |

**Additional file 9** Satisfaction with itch medication (LIS question 7). Results are scored out of 100 (maximum satisfaction) and are presented as mean (sd) for index and all reviews.
